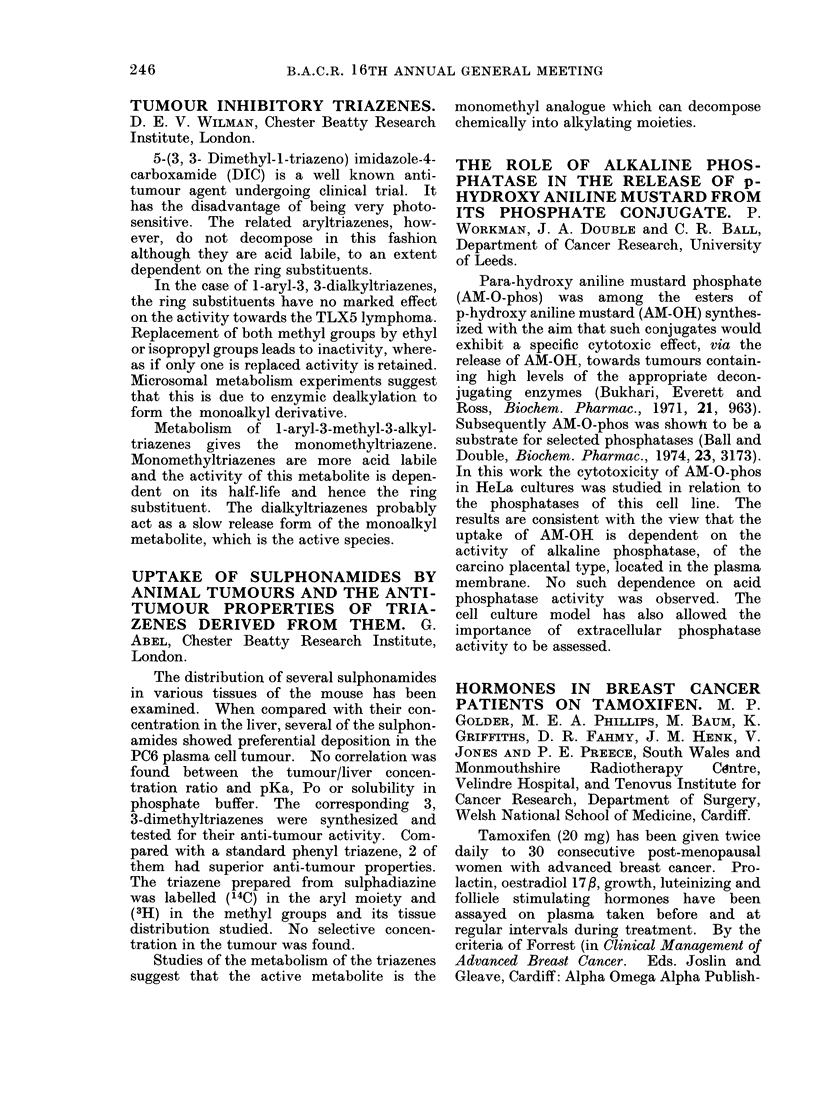# Proceedings: Uptake of sulphonamides by animal tumours and the anti-tumour properties of triazenes derived from them.

**DOI:** 10.1038/bjc.1975.174

**Published:** 1975-08

**Authors:** G. Abel


**UPTAKE OF SULPHONAMIDES BY ANIMAL TUMOURS AND THE ANTI-TUMOUR PROPERTIES OF TRIAZENES DERIVED FROM THEM.** G. ABEL, Chester Beatty Research Institute, London.

The distribution of several sulphonamides in various tissues of the mouse has been examined. When compared with their concentration in the liver, several of the sulphonamides showed preferential deposition in the PC6 plasma cell tumour. No correlation was found between the tumour/liver concentration ratio and pKa, Po or solubility in phosphate buffer. The corresponding 3, 3-dimethyltriazenes were synthesized and tested for their anti-tumour activity. Compared with a standard phenyl triazene, 2 of them had superior anti-tumour properties. The triazene prepared from sulphadiazine was labelled ($^{14}C$) in the aryl moiety and ($^{3}H$) in the methyl groups and its tissue distribution studied. No selective concentration in the tumour was found.

Studies of the metabolism of the triazenes suggest that the active metabolite is the monomethyl analogue which can decompose chemically into alkylating moieties.